# Development of genetically engineered iNKT cells expressing TCRs specific for the *M. tuberculosis* 38-kDa antigen

**DOI:** 10.1186/s12967-015-0502-4

**Published:** 2015-05-07

**Authors:** Zhen-Min Jiang, Wei Luo, Qian Wen, Su-Dong Liu, Pei-Pei Hao, Chao-Ying Zhou, Ming-Qian Zhou, Li Ma

**Affiliations:** Institute of Molecular Immunology, School of Biotechnology, Southern Medical University, Guangzhou, 510515 China

**Keywords:** Mycobacterium tuberculosis (Mtb), iNKT, T cell receptor (TCR), 38-kDa antigen, Gene-modification

## Abstract

**Introduction:**

The invariant natural killer T (iNKT) cell has been shown to play a central role in early stages immune responses against *Mycobacterium tuberculosis* (Mtb) infection, which become nonresponsive (anergic) and fails to control the growth of Mtb in patients with active tuberculosis. Enhancement of iNKT cell responses to Mtb antigens can help to resist infection.

**Study design and methods:**

In the present study, an Mtb 38-kDa antigen-specific T cell receptor (TCR) was isolated from human CD8^+^ T cells stimulated by 38-kDa antigen *in vitro*, and then transduced into primary iNKT cells by retrovirus vector.

**Results:**

The TCR gene-modified iNKT cells are endowed with new features to behave as a conventional MHC class I restricted CD8^+^ T lymphocyte by displaying specific antigen recognition and anti-Mtb antigen activity *in vitro*. At the same time, the engineered iNKT cells retaining its original capacity to be stimulated proliferation by non-protein antigens α-Gal-Cer.

**Conclusions:**

This work is the first attempt to engineer iNKT cells by exogenous TCR genes and demonstrated that iNKT cell, as well as CD4^+^ and CD8^+^ T cells, can be genetically engineered to confer them a defined and alternative specificity, which provides new insights into TCR gene therapy for tuberculosis patients, especially those infected with drug-resistant Mtb.

## Introduction

*Mycobacterium tuberculosis* (Mtb) is an intracellular bacterium that can be systemically cleared following a coordinated response between the innate and adaptive arms of the immune system. Invariant natural killer T (iNKT) cells, a sublineage of T lymphocytes with features of both T and natural killer (NK) cells, serve as a bridge between innate and adaptive immunity [1], act as the first line of defense against Mtb infectious. The iNKT cell number and interferon gamma (IFN-γ) production have been shown to peak as early as at 7 days post-Mtb infection [2], 1 to 2 weeks before the development of the MHC-restricted T cell response [3]. iNKT cells are recruited to infected lungs and killed intracellular Mtb either directly by granule-dependent mechanisms, or indirectly by secreting IFN-γ and tumor necrosis factor alpha (TNF-α) to activate infected macrophages [4,5]. Furthermore, iNKT cells were found to be indispensable at the earliest stage of granulomatous responses for effectively restricting Mtb dissemination [6-8]. Thus, iNKT cells have a central role in the early immune responses against Mtb infection. Although T cell receptors (TCRs) gene transfer is a widely used, mature technology, the successful development of TCR gene-modified iNKT cells has not been reported.

Although the number of iNKT cells increased in lesions of virulent Mtb infected mice, they become anergic and fail to control Mtb infection [9]. Additionally, the iNKT cell levels in peripheral blood mononuclear cells (PBMCs) of patients with chronic pulmonary Mtb infection are lower than that of both Mtb-exposed subjects and healthy donors [10]. Therefore, enhancement antibacterial activity of iNKT cells may be a promising strategy to suppress Mtb growth in the early stage of infection.

The emergences of multidrug-resistant strains and extensively drug-resistant strains make it urgent to designing immune therapeutic options to control TB [11]. Immunotherapy tuberculosis (TB) based on iNKT cells show great initial promise. Sada-Ovalle *et al.* transferred iNKT cells into a virulent Mtb-infected mouse model and found a significant reduction of pulmonary Mtb burden [12]. TCRs express on the surface of T lymphocytes that is responsible for recognizing antigens. TCR gene transfer is an attractive and powerful strategy to generate a large number of effector cells with high functional avidity in a short time [13]. Recently, TCR gene engineered T cells have been developed for adoptive cellular immune therapy of viral infectious diseases [14,15] and cancer [16]. Rosenberg *et al.* adoptively transferred engineered T cells carrying melanoma antigen-specific TCR genes to melanoma patients and achieved disease regression, demonstrating the potential clinical application value of this approach [17]. Although TCR gene transfer is widely used in conventional MHC class I or II restricted CD4 or CD8 T lymphocyte, engineered iNKT cells have never been reported.

Mtb 38-kDa antigen is one of the most immunogenic Mtb antigens that can be either secreted or expressed on the cell surface, evoking both prominent cellular and humoral immune responses [18]. 38-kDa antigen strongly polarized Th1 type immune response in vaccinated mice, which act as Bacillus Calmette Guérin (BCG), leading to significant reduction of bacterial load [19]. Furthermore, 38-kDa antigen has been used in the diagnosis of infection by displays higher specificity than other Mtb antigens [20,21].

The goal of this work was to engineer iNKT cells with an exogenous Mtb peptide-specific TCR gene by retrovirus transduction. Our work provides a foundation for the application of TCR gene-modified iNKT cells for future adoptive cellular immunotherapy of TB, especially with drug-resistant Mtb infection.

## Materials and methods

### Isolation and culture of T cells and dendritic cells

Healthy fresh blood samples were obtained from a HLA-A*2402^+^ healthy volunteer after obtaining written informed consent. This protocol approved by the ethics committee of Southern Medical University. PBMCs were isolated and divided into several aliquots. The isolation procedure and culture of T cells and dendritic cells (DCs) were performed as previously described [22]. Monocytes were positively selected using CD14^+^ magnetic bead (miniMACS, Miltenyi Biotec, Gladbach, Germany) and were induced to differentiate into dendritic cells (DCs) by adding 500 U/mL interleukin-4 (IL-4) and 1000 U/mL of granulocyte macrophage-colony stimulating factor (GM-CSF; both from PeproTech, Rocky Hill, NJ, USA), or into macrophages by adding 1000 U/mL of GM-CSF in RPMI-1640 (Corning, NY, USA). Both cells were cultured for 7 days and the medium was half-changed every 3 days.

### Stimulation of 38-kDa antigen-specific CD8^+^ T cell

On day 7 of DC culture, 10 μg/mL of the 38-kDa antigen (ImmunoDiagnostics, Woburn, MA, USA) and 20 ng/mL TNF-α were added. After 24 h the antigen-loaded DCs were cocultured with autologous CD8^+^ T cells sorting from PBMCs by CD8^+^ magnetic bead (miniMACS) at a responder to stimulator ratio of 10:1. The medium supplemented with 100 U/mL of IL-2 was half-changed on day 3. Such stimulation was repeated twice at 7-day intervals.

### TCR analysis of 38-kDa antigen-specific CD8^+^ T cell

To determine the TCR α and β chains of the 38-kDa antigen-specific CD8^+^ T cell, GeneScan analysis of TCR complementarity determining region 3 (CDR3) spectratyping was performed as previously described [23]. CD8^+^ T cells stimulated by 38-kDa antigen were immediately lysed. The RNA was extracted using the E.Z.N.A.® Total RNA Kit (OMEGA Bio-tek, Inc., Norcross, GA) and reverse transcription was performed using the RevertAid™ First Strand cDNA Synthesis Kit (Fermentas, Life Sciences, Ontario, Canada) according to the manufactures’ instruction.

CDR3 analysis within the TCR α chain was performed by PCR amplification each of the 32 human TCR AV gene families, and the TCR β chain was performed by amplification of 24 BV gene families. These were carried out in a volume of 25 μl containing 1 μl each of the forward AV or BV primer and the reverse AC-FAM or BC-FAM primer, 1 mM MgCl_2_, 5 mM Tris–HCl, 100 mM of each dNTP, and 0.5 μl of cDNA. PCR amplification for TCR α chain was carried out with 35 cycles of denaturing at 95°C for 30 s, annealing at 60°C for 30s, and extension at 72°C for 30 s, and amplification for β chain was carried out with 35 cycles of denaturing at 94°C for 30 s, annealing at 55°C for 30s, and extension at 72°C for 30 s. Two μl Fluorescent PCR products mixed with 2 μl of formamide, 0.5 μl of loading dye (25 mM EDTA, 50 ng/ml blue dextran), 0.5 μl of GeneScan-500 TAMRA dye-labeled size standards, the mixture was denatured at 95°C for 2 min, and 2 μl was loaded onto the gel (6% acrylamide, 6 M urea sequencing gel) and run for 2 h on 373A DNA sequencer (Applied Biosystems, USA), DNA products of the appropriate lengths were analyzed using GeneScan software version 672. The relative fluorescence intensity (RI) was measured as: RI = 100 × (clonal peak area) / (total peak area). The following criteria were used to determine whether a given TCR AV or BV family was monoclonal proliferation: a single peak with an RI% greater than 35% [24].

### Generation of mutated TCRs

According to the monoclonal expansion antigen specific CD8^+^ TCR gene families identified by GeneScan analysis, primers were designed to amplify the full-length TCR α9 (Accession No.: NG_001332.2) and β5 (Accession No.: NG_001333.2) coding sequences incorporating the nine amino acid-mutated C regions using overlapping PCR methods [25]. The β5-chain V region (and a portion of the C region) was amplified with the β5 forward primer P1 5’-CCGGAATTCATGGGCTCCAGGCTGCTCTGTTGGGTGCTGCTTTG-3’ and the reverse primer P2 5’-CTTGTTCAGGTCCTCTACAACTGTGAGTCTGGTGCCTTGT-3’. Amplification of the mutated C regions (and a portion of the V region) were performed with the forward primer P3 5’-AGACTCACAGTTGTAGAGGACCTGAACAAGGTGTTCCCA C-3’ and the reverse primer P4 [containing 5’-end of P2A [26].] 5’-CTTCCACGTCTCCTGCTTGCTTTAACAGAGAGAAGTTCGTGGCTCCGGAGCCGAATCCTTTCTCTTGACCATGGCCAT-3’. A second PCR using the primers P1 and P4 completed the β5-chain. Similarly, the α9-chain was generated with the following primers: the α9 forward primer P5 (containing 3’-end of P2A) 5’-CGAACTTCTCTCTGTTAAAGCAAGCAGGAGACGTGGAAGAAAACCCCGGTCCCATGTGGGGAGCTTTCCTTCTCTATGT-3’ with the reverse primer P6 5’-GTCAGGGTTCTGGATATTTGCAATCACAGAAAGTCTTGTG-3’, the forward primer P7 5’-TCTGTGATTGCAAATATCCAGAACCCTGACCCTGCCGTGT-3’ with the reverse primer P8 5’-CCGCTCGAGTCAGCTGGACCACAGCCGCAGCGTCATGAGCAG-3’. The α9-chain was completed with the primers P5 and P8.

Subsequently, a third PCR using the primers P1 and P8 completed the full-length TCR β5/α9 in which the two TCR chains were linked with the P2A peptide sequence. The PCR products were digested with BamH-I and Xho-I (TaKaRa, Japan) and inserted into the pMX-internal ribosomal entry site (IRES)-green fluorescent protein (GFP) retroviral vector (kindly provided by Han H, Fourth Military Medical University, Xi’an, China).

### Construction of recombinant retroviral vector

To prevent mispairing of the exogenous and endogenous TCR, α9 and β5 chains contain 9 amino acid-mutated C regions amplify by the above overlapping PCR methods have been connected by 2A and inserted into the retrovirus empty vector PMX-IRES-GFP. Construction of the recombinant retroviral vector was performed as previously described [25]. Retrovirus was packaged by co-transfection of the recombinant vector pMX-β5-2A-α9 and the VSV-G envelope protein vector into the retroviral packaging cell line GP2-293 with Lipofectamine 2000 Transduction Reagent (Invitrogen, Carlsbad, CA, USA) following manufacturer’s instructions and concentrated and the virus titer was determined as described before [25].

### Culture and isolation of iNKT cells

One aliquot of PBMCs was inoculated in 24-well plates (Nunc, Roskilde, Demark) at 2 × 10^6^/well in RPMI-1640 medium (Hyclone Ltd, Logan, UT) containing 10% fetal bovine serum (FBS; Hyclone), 100 ng/mL α-GalCer (Kirin Brewery, Gunma, Japan) and 10 ng/mL IL-2 (PeproTech, Rocky Hill, NJ, USA) for 7 days. For enrichment and expansion of iNKT cells, the culture of another aliquot of PBMCs (used as autologous APCs) was supplemented with 500 U/mL IL-4 and 1000 U/mL GM-CSF for 7 d and pulsed with 100 ng/mL a-GalCer and 20 ng/mL TNF-α(PeproTech, Rocky Hill, NJ, USA) for 24 h, and then added at 7-d interval into the iNKT culture. Medium was half-changed every 2–3 days. After the second stimulation, iNKT cells were positively selected using Vα24^+^ TCR magnetic bead (miniMACS, Miltenyi Biotec, Gladbach, Germany).

### Transduction of iNKT cells

iNKT cells (1 × 10^6^ cells/mL) were seeded in the presence of 10 ng/mL IL-2 and 50 ng/mL anti-human CD3/CD28 Dynabeads (Invitrogen) 48 h prior to transduction. The concentrated retroviral supernatant containing 8 μg/mL of polybrene (Sigma, St Louis, MO, USA) was added and 4 h later the same fresh medium was supplemented to dilute the polybrene to 2 mg/mL. Five days after transduction, cells were collected and labeled with anti-TCR Vβ5 antibody to assess the transduction efficiency and the expression of exogenous TCR Vβ5 by Flow cytometry.

### Immunofluorescence

Transduced or untransduced iNKT cells (1 × 10^6^) were stained with the following antibodies using the method described before [22]: phycoerythrin (PE)-TCR Vα24 (clone 6B11, Miltenyi Biotec), allophycocyanin (APC)-TCR Vβ5 (Immudex), APC- or PE-labeled IgG1 (Becton Dickinson Pharmingen, San Jose, CA, USA). Expression of exogenous genes was observed using a confocal laser scanning microscope (FV1000, Olympus, Tokyo, Japan) and quantified by Flow cytometry on a FACSCalibur flow cytometer using CELLQuest software (Becton Dickinson).

Intracellular Flow was used to further detail the iNKT cell populations expressed cytokines upon activated by Mtb antigen 38-kDa stimulation. TCR gene-modified iNKT cells were stimulated by 38-kDa antigen-loaded DCs (E/T = 20). After 24 h, 1 × 10^6^ of cocultured cells were centrifugated at 2000 rpm for 5 min and washed by PBS for 3 times, then fixed and permeabilized with BD Cytofix/Cytoperm™ Fixation/Permeabilization Solution Kit (BD Pharmingen Company, San Jose, CA, USA) and stained by BD FastImmune fluorescein isothiocyanate (FITC) -labeled Anti-IFN-γ antibody (Becton Dickinson, San Diego, CA, USA) according to the instructions, expression of IFN-γ was detected by Flow cytometry.

### Cell proliferation assays

Irradiated (30 Gy) DCs were cocultured at 5 × 10^3^ cells/well in triplicate with autologous iNKT cells in a 96-well plate at an effector/target (E/T) ratio of 20:1. After culturing for 1, 4, or 7 days, cell proliferation was measured using the Cell Counting Kit-8 (Dojindo, Kumamoto, Japan) according to the instructions of manufacturers. The absorbance was determined with a spectral scanning multimode reader (Scientific Varioskan Flash, ThermoElectron, San Jose, CA, USA).

### Cytokine release assay

Antigen-loaded autologous DCs (5 × 10^3^ cells/well) that were irradiated at a dosage of 30 Gy were cocultured with iNKT cells as stimulators in 96-well plates at the ratios as indicated in the legends. Also at the indicated time the culture supernatants were collected for assay of IFN-γ, TNF-α, or granzyme B (GrB) release with ELISA (Bender MedSystems, Vienna, Austria) as per the manufacturer’s instructions.

### Cytotoxicity assays

Cytotoxicity assays were carried out using a DELFIA EuTDA cytotoxicity kit (Perkin-Elmer Life Sciences, Norwalk, CT, USA) according to the manufacturers’ instruction. Eu-labeled autologous DCs (5 × 10^3^) were co-cultured with TCR-transduced effector cells at an E/T ratio of 30:1. After co-cultured 4 h, the specific lysis was detected with the Varioskan Flash reader (ThermoElectron) and calculated using the following formula: [(experimental release - spontaneous release)/(maximum release - spontaneous release)] × 100, where the maximum or the spontaneous release were determined by detecting the target cells incubated alone with or without complete cytolysis.

### Statistical analysis

One-way analysis of variance was used to test the statistical significance. Welch correction was applied for the unequal variance. Least significant difference multiple comparison test or Dunnett’s T3 was used to compare the means between groups. Two-sided *P*-values < 0.05 were considered statistically significant. Statistical analyses were performed using the SPSS for windows statistical package version 17.0 (SPSS, Chicago, IL, USA).

## Results

### Screening for monoclonal expansion TCR gene families

**Figure 1:**
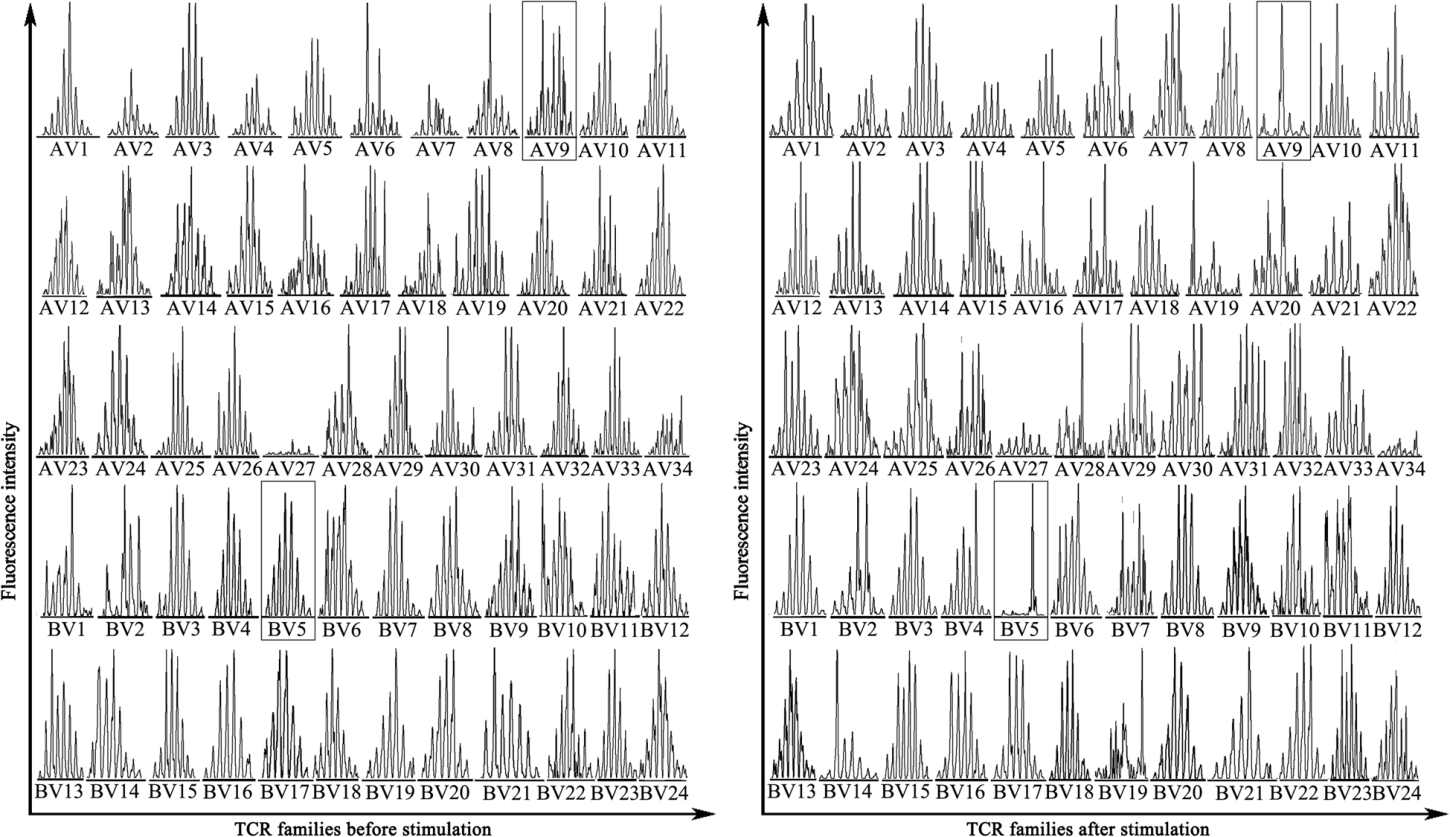
CDR3 spectratyping of CD8^+^ T cell TCR α and β chain genes before and after stimulation with the Mtb 38-kDa antigen. The polyclonal Gaussian distribution of Vβ5 and Vα9 gene families (framed) before stimulation changed to a monoclonal proliferation peak after stimulation, indicating their specificity to the Mtb 38-kDa antigen.

The CDR3 spectratyping of almost all TCR Vα and Vβ gene families of CD8^+^ T cells displayed a Gaussian distribution of the expected polyclonal T cell populations before stimulation. However, several families such as TCR Vα9, Vα19 and Vβ5 showed abnormal distribution characteristic of oligoclonal or monoclonal expansion after stimulation. The TCR gene families showed a single peak and RI% > 35% after stimulation indicated monoclonal expansion and specific to the Mtb 38-kDa antigen, therefore the TCR Vα9 and Vβ5 gene families was selected (Figure 1).

### Retroviral vector construction

**Figure 2:**
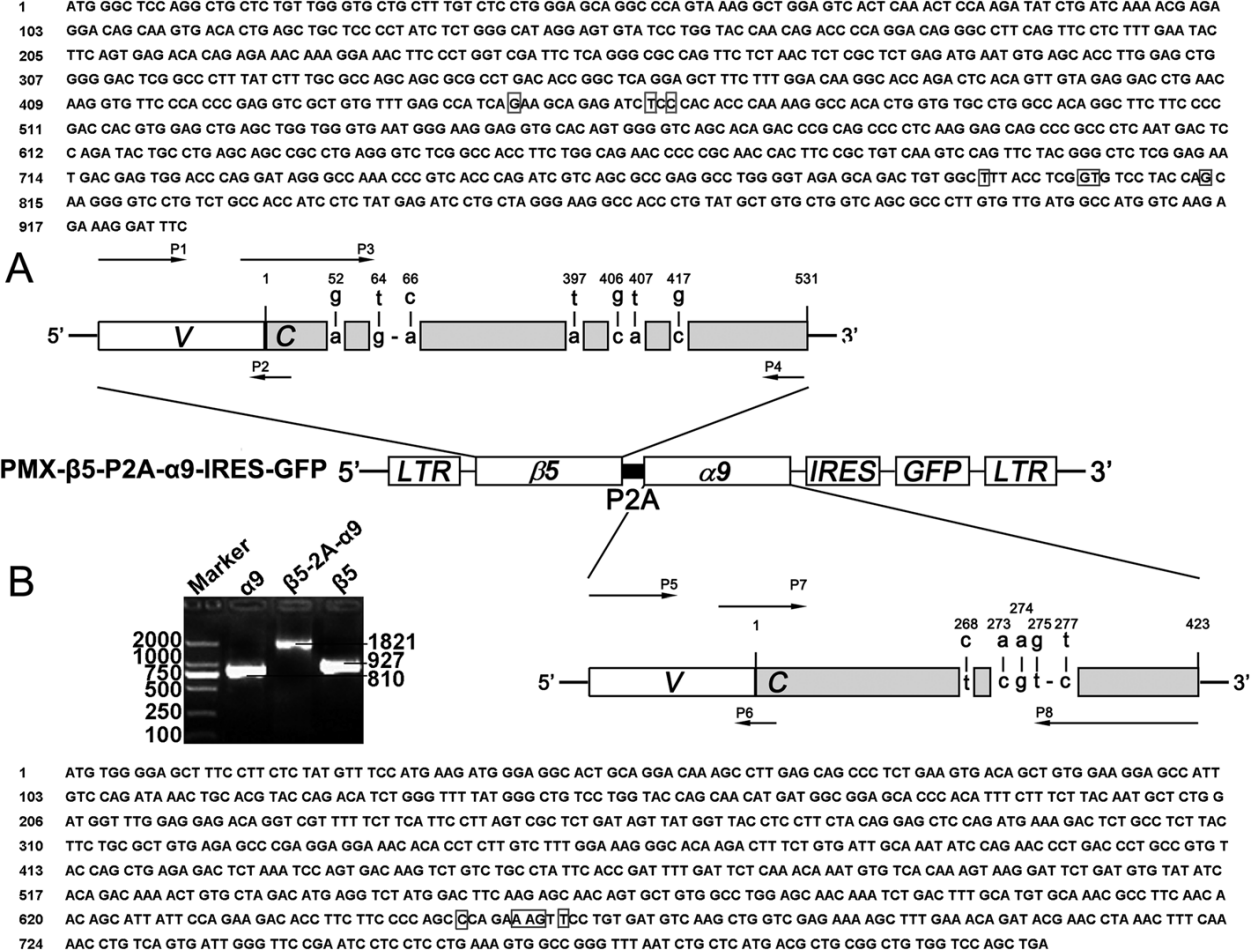
Schematic representation of the recombinant retroviral vector. **(A)** The sequence of wild type TCR β5 (the above panel) and α9 genes (the below panel), and the structure diagram of recombinant retroviral vector pMX-β5-P2A-α9-IRES-GFP carrying the hybrid TCR β5 and α9 genes with point mutations in the C regions (the middle panel). **(B)** The PCR results analyzed with 2% agarose gel showed the full-length genes of hybrid β5-P2A-α9, β5 and α9 chains, respectively.

The sequences of full-length TCR β5 (927 bp) and α9 (810 bp) gene were shown in Figure 2A. The mutated TCR β5 and α9 gene incorporating the nine amino acid-mutated C regions was created using overlapping PCR methods. Sequencing results showed no undesired mutation in the recombinant DNA fragment (Data not shown). The recombinant retroviral vector pMX-β5-P2A-α9-IRES-GFP was constructed by inserting the full-length TCR gene β5-P2A-α9 (1821 bp) into the empty vector PMX-IRES-GFP (Figure 2A), and was then used to package the recombinant retrovirus. According to Flow cytometry analysis, the retrovirus titer was approximately 1.2 × 10^7^ IU/mL (Data not shown).

### Efficient expansion and isolation of human iNKT cells

**Figure 3:**
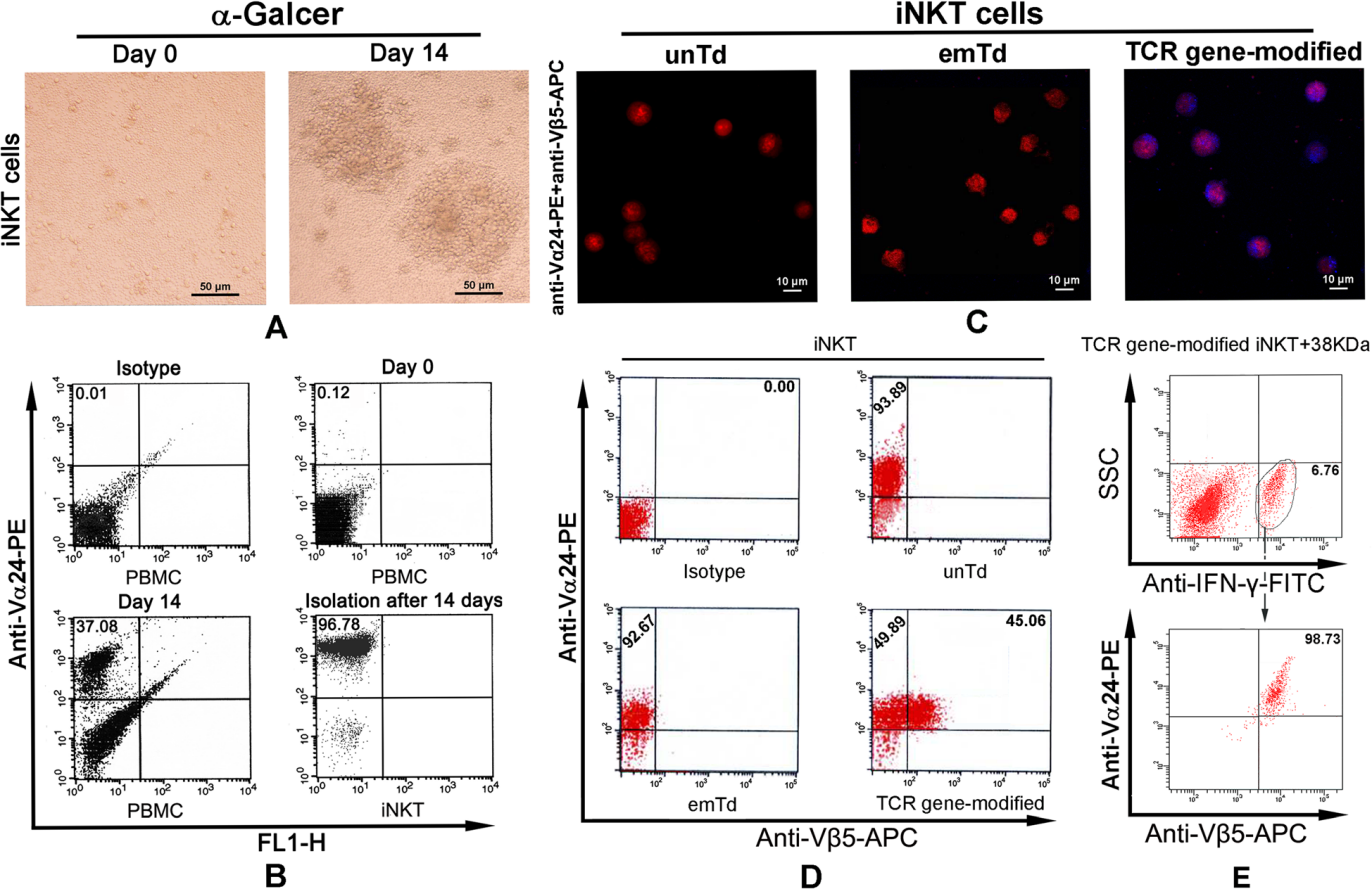
Culture and transduction of human iNKT cells. **(A)** iNKT cells in PBMCs were expanded 300-folds after stimulation with α-GalCer for 14 d. The upper panel: prominent cell colonies were observed (Magnification: 200×); the lower panel: the percentage of iNKT cells in PBMCs and the purity of iNKT cells after isolation with microbeads. **(B)** The purity of iNKT cells before and after isolation with microbeads, **(C)** and the expression of the PE-Vα24 (red) and APC-Vβ5 (blue) genes after retroviral transduction, were observed under fluorescence microscopy **(D)** and analyzed with Flow cytometry. **(E)** Intracellular Flow cytometry analysis the iNKT cell populations expressed IFN-γ upon activated by Mtb antigen 38-kDa stimulation. UnTd (untransduced iNKT cells); EmTd (empty vector-transduced iNKT cells); Td (TCR gene-modified iNKT cells). Td + 38-kDa (TCR gene-modified iNKT cells + 38-kDa antigen-loaded DCs).

The percentage of iNKT cells in human PBMCs is extremely low (0.01% - 1%). Short-term *in vitro* culture to obtain sufficient numbers of cells is indispensable for iNKT cell studies. Under the microscope, iNKT cells in human PBMCs cultured using whole PBMCs as APCs to presented alpha-Gal-cer antigen showing obvious, bulky, and numerous colonies in day 14 (Figure 3A). After isolation by positive magnetic bead sorting, the purity of Vα24^+^ iNKT cells determined by Flow cytometry was achieved 96.78% (Figure 3B).

### Expression of exogenous genes by TCR gene-modified iNKT cells

For functional avidity, it is important that the expression level of the transduced TCR gene be high on the recipient cell surface. Under fluorescence microscopy, the APC fluorescence of TCR Vβ5 labeling was clearly observed in TCR gene-modified iNKT cells (Td), but not in untransduced (UnTd) and empty vector-transduced (EmTd) iNKT cells, 5 d after transduction (Figure 3C). Flow cytometry analysis demonstrated that 45.06% of TCR gene-modified iNKT cells express both Vα24 and Vβ5. By contrast, untransduced and empty vector-transduced iNKT cell controls have no Vβ5 expression (Figure 3D).

### Antigen-specific proliferation of TCR-engineered iNKT cells

Significant proliferation was seen in TCR gene-modified iNKT cells stimulated with 38-kDa loaded DCs (*P* < 0.001) at days 4 and 7 after stimulation, suggesting that TCR gene-modified iNKT cells proliferated rapidly upon 38-kDa antigen stimulation. For the same engineered iNKT cells, stimulation with unassociated antigen OVA and Mtb-associated antigen ESAT-6 did not induce obvious proliferation, similar to untransduced or empty vector-transduced iNKT cells stimulated with 38-kDa antigen. Obvious proliferation was also observed in engineered iNKT cells stimulated by alpha-Gal-cer, demonstrated that the engineered iNKT cells retaining its original capacity to be stimulated proliferation by non-proteic antigens alpha-Gal-Cer.

**Figure 4:**
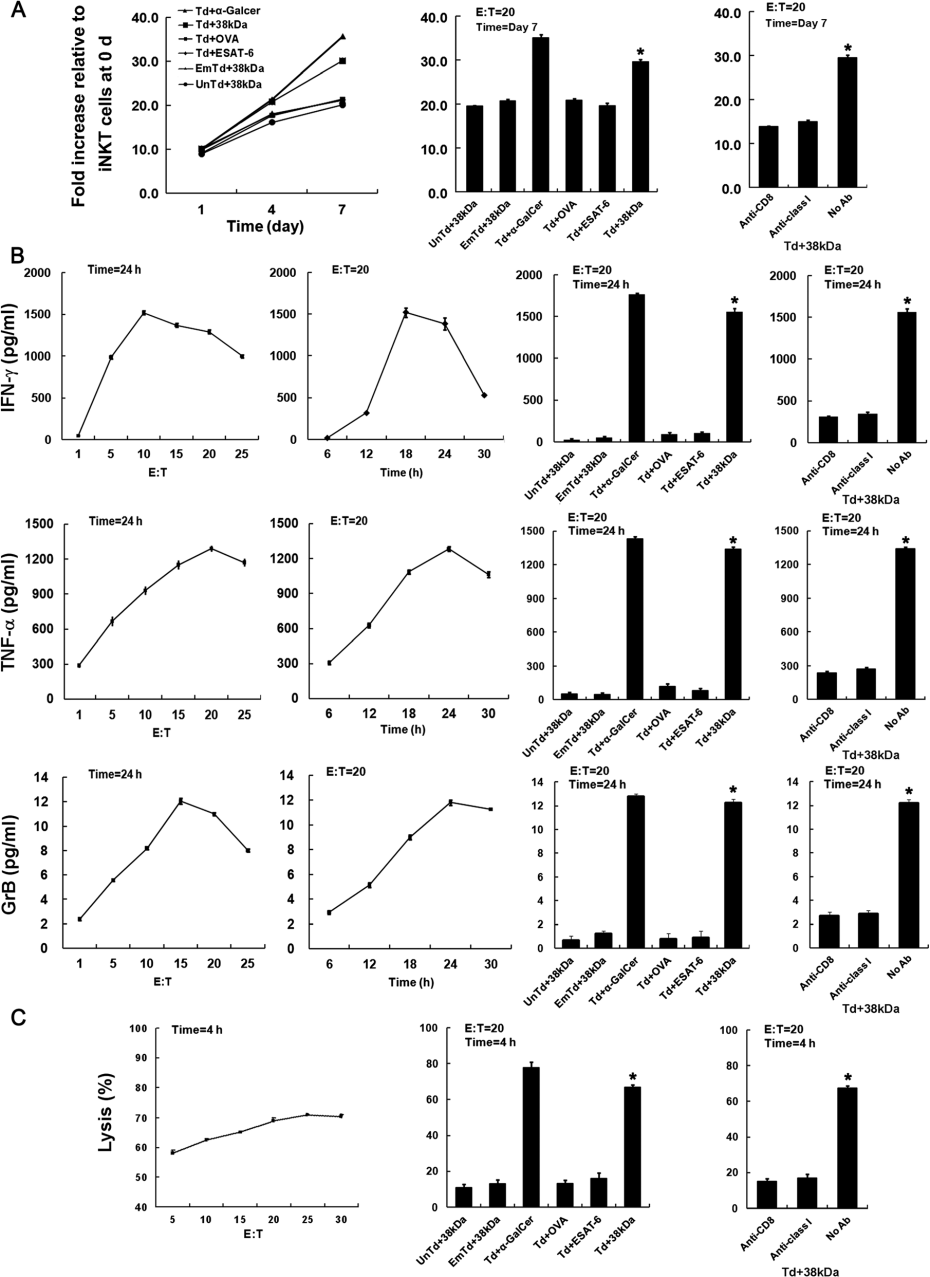
Proliferation, cytokine secretion and cytotoxicity of TCR gene-modified iNKT cells. **(A)** Proliferation of TCR gene-modified iNKT cells co-cultured with antigen-loaded or -unloaded DCs. **(B)** INF-γ, TNF-α and GrB secretion of TCR gene-modified iNKT cells at different E:T ratios (left panels) and time points (center panel). Compared with other control groups, TCR gene-modified iNKT cells exerted enhanced specific anti-TB antigen activities and these activities can be block by anti-HLA and anti-CD8 antibody (right panels). **(C)** Cytolytic activity of exogenous TCR gene-modified iNKT cells. UnTd + 38-kDa (untransduced iNKT cells + 38-kDa antigen-loaded DCs); EmTd + 38-kDa (empty vector- transduced iNKT cells + 38-kDa antigen-loaded DCs); Td + α-Galcer (TCR gene-modified iNKT cells + α-Gal-cer-loaded DCs); Td + OVA (TCR gene-modified iNKT cells + OVA-loaded DCs); Td + ESAT-6 (TCR gene-modified iNKT cells + ESAT-6 antigen -loaded DCs); Td + 38-kDa (TCR gene-modified iNKT cells + 38-kDa antigen-loaded DCs). *P < 0.001 compared with UnTd + 38-kDa group.

The proliferation response of TCR gene-modified iNKT cells stimulated by 38-kDa can been blocked by both anti-CD8 and anti-HLA Class I antibodies (Figure 4A), demonstrated that TCR gene-modification of iNKT cells can lead to highly specific proliferation activity.

### Antigen-specific IFN-γ, TNF-α, and GrB production in TCR gene-modified iNKT cells

TCR gene-modified iNKT cells stimulated by 38-kDa antigen-loaded DCs produced significantly higher levels of cytokine IFN-γ (*P* < 0.001), TNF-α (*P* < 0.001), and granzyme B (GrB; *P* < 0.001) than the similarly stimulated negative controls (untransduced and empty vector-transduced iNKT cells stimulated by 38-kDa antigen-loaded DCs), Compared with non-specific antigen controls (the TCR gene-modified iNKT cells stimulated by OVA- and ESAT-6 loaded DCs), the levels of IFN-γ (*P* < 0.001), TNF-α (*P* < 0.001), and GrB (*P* < 0.001) expressed by the same type of effector cells stimulated by 38-kDa antigen-loaded DCs were also higher (Figure 4B).

To identify if the TCR-transduced iNKT cells were indeed the responders, an assay of intracellular IFN-γ staining have been performed on engineered iNKT cells stimulated by 38-kDa antigen-loaded DCs, Flow cytometry results showing that 98.73% of IFN-γ producing cells are exogenous TCR gene expressed iNKT cells (Figure 3E). Moreover, when anti-HLA Class I or anti-CD8 antibodies was added, the amounts of cytokines in the TCR gene-modified iNKT cells stimulated by 38-kDa antigen-loaded DCs were obviously decrease (Figure 4B), suggesting that TCR gene-modified iNKT cells were endowed with functional avidity in responded specifically to 38-kDa antigen stimulation.

### Antigen-specific cytolytic activity of TCR gene-modified iNKT cells

Similar to the cytolytic activity of iNKT cells stimulated by alpha-Gal-cer, TCR gene-modified iNKT cells co-cultured with 38-kDa antigen-loaded DCs had a high cytolytic activity. Almost 70% of 38-kDa antigen-loaded DCs were specifically lysed by TCR gene-modified iNKT cells at an E/T ratio of 30:1. By contrast, untransduced and empty vector-transduced iNKT cells had only about 10% lytic activity, respectively. The lytic activity of TCR gene-modified iNKT cells stimulated with 38-kDa antigen was significantly higher than those stimulated with OVA and ESAT-6 antigens (*P* < 0.001). Moreover, the lytic activity of the TCR gene-modified iNKT cells response to 38-kDa antigen-loaded DCs were obviously decrease when anti-MHC Class I or anti-CD8 antibodies was used to blocking the costimulatory signal of specific recognize (Figure 4C), suggesting that TCR gene-modified iNKT cells were endowed with specifically lysed avidity.

## Discussion

Although current pharmacotherapy is effective against susceptible Mtb strains, the emergence of multidrug and extensively drug-resistant strains [27] requires the design of new therapeutic options to enhance immune response. iNKT cells are involved in various immune reactions in early TB infection, such as regulation of innate and acquired immune responses [3,28]. However, it was reported that the number and function of iNKT cell selectively reduced in PBMCs of pulmonary TB patients, and increased to the pre-infection level following effective treatments and disease remission [29,30]. Consistent with other impaired immune responses in active pulmonary TB patients, iNKT cell deficiency may contribute to Mtb replication and diffusion [31]. In this pilot study, we established TCR gene-modified iNKT cells that specifically recognized Mtb antigenic, and displayed increased functional avidity compared with unmodified iNKT cells.

TCR gene transfer is an attractive strategy to obtain antigen-specific effector cells in a short time. However, a likely difficulty with this approach is the potential mismatch of endogenous and exogenous TCR genes: that is to say, the introduced α or β chains may be mispaired with the endogenous α or β chains. As a result of this situation, both the expression and the function of the exogenous Mtb antigen-specific TCR genes on the recipient cell surface will be decreased. To minimize the likelihood of gene mismatch and to enhance the stability of the exogenous TCRs, we introduced several mutations into the TCR genes. Nine amino acids in the C regions of the exogenous α and β chains were substituted with their homologous counterparts in the murine sequence [32]. A P2A ribosomal skip element was used to link the exogenous α and β chain genes to achieve equimolar expression [26]. Exogenous genes constructed by these stactics were expressed normally, which is consistent with previous reports [33].

Human iNKT cells express an invariant Vα24Jα18/Vβ11 TCR that only recognizes glycolipid antigens in conjunction with the CD1d molecule [34]. Genetic engineering with HLA-restricted TCRs endows iNKT cells with the specific recognition of the antigenic presented by autologous or allogeneic APCs, one feature of the adaptive immune system. Thus, to avoid cell rejection, the prospective iNKT-based adoptive immunotherapy must be restricted to TB patients who are HLA-matching with the iNKT cells. In this study, the HLA type HLA-A*2402 was chosen for its highest proportion (>50%) in the Chinese population. Despite the HLA restrictions in adoptive immunotherapy, this TCR gene transfer strategy can be widely used in any condition of HLA restriction.

After recognizing the specific antigen presented by autologous DCs, TCR gene-modified iNKT cells specifically killed Mtb antigen-loaded target cells directly by granule-dependent mechanisms, and simultaneously exhibited an enhanced regulating function in the early exposure of the Mtb antigen peptide through cytokine secretion. Further studies should evaluate the efficacy of TCR gene-modified iNKT cells in Mtb-infected animal models. The iNKT cells transferred through tail vein injection should move into the infected lung through chemotaxis, via the interaction between CCR4 on the iNKT cells and thymus- and activation-regulated chemokines [35]. This process would greatly enhance the possibility of the recognition between the iNKT cells and the infected target cells.

## Conclusions

We modified human iNKT cells by transduction with a recombinant retrovirus carrying Mtb antigen-specific TCR genes. The TCR genes were successfully expressed on the iNKT cell surface and endowed the modified cells with significantly enhanced anti-Mtb antigen activity. To the best of our knowledge, this is the first attempt to engineer iNKT cells by exogenous TCR genes. By introducing TCR gene-modified iNKT cells, our results provide new insights into TCR gene therapy and lay a basis for future adoptive immunotherapy designed to prevent or treat drug-resistant TB, particularly in immunocompromised persons.

## Abbreviations

Mtb: Mycobacterium tuberculosis
iNKT: Invariant natural killer T
NK: Natural killer
IFN-γ: Interferon gamma
TNF-α: Tumor necrosis factor alpha
PBMCs: Peripheral blood mononuclear cells
TB: Tuberculosis
TCRs: T cell receptors
BCG: Bacillus Calmette Guérin
DCs: Dendritic cells
IL-4: Interleukin-4
GM-CSF: Granulocyte macrophage colony stimulating factor
PE: Phycoerythrin
APC: Allophycocyanin
FITC: Fluorescein isothiocyanate
CDR3: Complementarity determining region 3
GrB: Granzyme B
IRES: Internal ribosomal entry site
GFP: Green fluorescent protein

## References

[1] Cerundolo V, Kronenberg M (2010). The role of invariant NKT cells at the interface of innate and adaptive immunity. Semin Immunol.

[2] Mogues T, Goodrich ME, Ryan L, LaCourse R, North RJ (2010). The relative importance of T cell subsets in immunity and immunopathology of airborne Mycobacterium tuberculosis infection in mice. J Exp Med.

[3] Dieli F, Taniguchi M, Kronenberg M, Sidobre S, Ivanyi J, Fattorini L (2003). An anti-inflammatory role for V alpha 14 NK T cells in Mycobacterium bovis bacillus Calmette-Guérin-infected mice. J Immunol.

[4] Gansert JL, Kiessler V, Engele M, Wittke F, Röllinghoff M, Krensky AM (2003). Human NKT cells express granulysin and exhibit antimycobacterial activity. J Immunol.

[5] Rijavec M, Volarevic S, Osolnik K, Kosnik M, Korosec P (2003). Natural killer T cells in pulmonary disorders. Respir Med.

[6] Mempel M, Ronet C, Suarez F, Gilleron M, Puzo G, Van Kaer L (2002). Natural killer T cells restricted by the monomorphic MHC class 1b CD1d1 molecules behave like inflammatory cells. J Immunol.

[7] Apostolou I, Takahama Y, Belmant C, Kawano T, Huerre M, Marchal G (1999). Murine natural killer T (NKT) cells [correction of natural killer cells] contribute to the granulomatous reaction caused by mycobacterial cell walls. Proc Natl Acad Sci U S A.

[8] Gilleron M, Ronet C, Mempel M, Monsarrat B, Gachelin G, Puzo G (2001). Acylation state of the phosphatidylinositol mannosides from Mycobacterium bovis bacillus Calmette Guérin and ability to induce granuloma and recruit natural killer T cells. J Biol Chem.

[9] Chiba A, Dascher CC, Besra GS, Brenner MB (2008). Rapid NKT cell responses are self-terminating during the course of microbial infection. J Immunol.

[10] Snyder-Cappione JE, Nixon DF, Loo CP, Chapman JM, Meiklejohn DA, Melo FF (2007). Individuals with pulmonary tuberculosis have lower levels of circulating CD1d-restricted NKT cells. J Infect Dis.

[11] Lange C, Grobusch MP, Wagner D (2008). Extensively drug-resistant tuberculosis. Dtsch Med Wochenschr.

[12] Sada-Ovalle I, Chiba A, Gonzales A, Brenner MB, Behar SM (2008). Innate invariant NKT cells recognize Mycobacterium tuberculosis-infected macrophages, produce interferon-gamma, and kill intracellular bacteria. PLoS Pathog.

[13] Schumacher TN (2002). T-cell-receptor gene therapy. Nat Rev Immunol.

[14] Kessels HW, Wolkers MC, van den Boom MD, van der Valk MA, Schumacher TN (2001). Immunotherapy through TCR gene transfer. Nat Immunol.

[15] Kitchen SG, Levin BR, Bristol G, Rezek V, Kim S, Aguilera-Sandoval C (2012). In Vivo Suppression of HIV by Antigen Specific T Cells Derived from Engineered Hematopoietic Stem Cells. PLoS Pathog.

[16] Nicholson E, Ghorashian S, Stauss H (2012). Improving TCR Gene Therapy for Treatment of Haematological Malignancies. Adv Hematol.

[17] Morgan RA, Dudley ME, Wunderlich JR, Hughes MS, Yang JC, Sherry RM (2006). Cancer regression in patients after transfer of genetically engineered lymphocytes. Science.

[18] Zhang SL, Zhao JW, Sun ZQ, Yang EZ, Yan JH, Zhao Q (2009). Development and evaluation of a novel multiple-antigen ELISA for serodiagnosis of tuberculosis. Tuberc Edinb.

[19] Zhu X, Venkataprasad N, Thangaraj HS, Hill M, Singh M, Ivanyi J (1997). Functions and specificity of T cells following nucleic acid vaccination of mice against Mycobacterium tuberculosis infection. J Immunol.

[20] Negi SS, Anand R, Pasha ST, Gupta S, Basir SF, Khare S (2007). Diagnostic potential of IS6110, 38 kDa, 65 kDa and 85B sequence-based polymerase chain reaction in the diagnosis of Mycobacterium tuberculosis in clinical samples. Indian J Med Microbiol.

[21] Uma Devi KR, Ramalingam B, Brennan PJ, Narayanan PR, Raja A (2001). Specific and early detection of IgG, IgA and IgM antibodies to Mycobacterium tuberculosis 38 kDa antigen in pulmonary tuberculosis. Tuberculosis (Edinb).

[22] Bohnenkamp HR, Noll T (2003). Development of a standardized protocol for reproducible generation of matured monocyte-derived dendritic cells suitable for clinical application. Cytotechnology.

[23] Luo W, Ma L, Wen Q, Wang N, Zhou MQ, Wang XN (2008). Analysis of the interindividual conservation of T cell receptor alpha- and beta-chain variable regions gene in the peripheral blood of patients with systemic lupus erythematosus. Clin Exp Immunol.

[24] Luo W, Ma L, Wen Q, Zhou MQ, Wang XN (2009). Analysis of the conservation of T cell receptor alpha and beta chain variable regions gene in pp 65 peptide-specific HLA-A*0201-restricted CD8+ T cells. Cell Mol Immunol.

[25] Luo W, Zhang XB, Huang YT, Hao PP, Jiang ZM, Wen Q (2011). Development of genetically engineered CD4^+^ and CD8^+^ T cells expressing TCRs specific for a M. tuberculosis 38-kDa antigen. J Mol Med.

[26] Hu T, Fu Q, Chen P, Zhang K, Guo D (2009). Generation of a stable mammalian cell line for simultaneous expression of multiple genes by using 2A peptide-based lentiviral vector. Biotechnol Lett.

[27] Jassal M, Bishai W (2009). Extensively drug-resistant tuberculosis. Lancet Infect Dis.

[28] Stolberg VR, Chiu BC, Martin BE, Shah SA, Sandor M, Chensue SW (2011). Cysteine-cysteinyl chemokine receptor 6 mediates invariant natural killer T cell airway recruitment and innate stage resistance during mycobacterial infection. J Innate Immun.

[29] Sutherland JS, Jeffries DJ, Donkor S, Walther B, Hill PC, Adetifa IM (2009). High granulocyte/lymphocyte ratio and paucity of NKT cells defines TB disease in a TB-endemic setting. Tuberculosis (Edinb).

[30] Veenstra H, Baumann R, Carroll NM, Lukey PT, Kidd M, Beyers N (2006). Changes in leucocyte and lymphocyte subsets during tuberculosis treatment; prominence of CD3dimCD56^+^ natural killer T cells in fast treatment responders. Clin Exp Immunol.

[31] Im JS, Kang TJ, Lee SB, Kim CH, Lee SH, Venkataswamy MM (2008). Alteration of the relative levels of iNKT cell subsets is associated with chronic mycobacterial infections. Clin Immunol.

[32] Bialer G, Horovitz-Fried M, Ya’acobi S, Morgan R, Cohen C (2010). Selected murine residues endow human TCR with enhanced tumor recognition. J Immunol.

[33] Leisegang M, Engels B, Meyerhuber P, Kieback E, Sommermeyer D, Xue SA (2008). Enhanced functionality of T cell receptor-redirected T cells is defined by the transgene cassette. J Mol Med (Berl).

[34] Borg NA, Wun KS, Kjer-Nielsen L, Wilce MC, Pellicci DG, Koh R (2007). CD1d-lipid-antigen recognition by the semi-invariant NKT T-cell receptor. Nature.

[35] Bourgeois EA, Levescot A, Diem S, Chauvineau A, Bergès H, Milpied P (2011). A natural protective function of invariant NKT cells in a mouse model of innate-cell-driven lung inflammation. Eur J Immunol.

